# Interfacial Modification and Bending Performance of 3D Orthogonal Woven Composites with Basalt Filament Yarns

**DOI:** 10.3390/ma16114015

**Published:** 2023-05-27

**Authors:** Lihua Lyu, Fangfang Wen, Tingting Lyu, Xinghai Zhou, Yuan Gao

**Affiliations:** School of Textile and Material Engineering, Dalian Polytechnic University, Dalian 116034, China

**Keywords:** 3D woven composites, basalt filament yarns, surface modification, mechanical properties, finite element simulation, damage mechanism

## Abstract

To improve their interfacial properties, 3D orthogonal woven fabrics with basalt filament yarns were modified with functionalized carboxylated carbon nanotubes (KH570-MWCNTs) and polydopamine (PDA). Fourier infrared spectroscopy (FT-IR) analysis and scanning electron microscopy (SEM) testing were used. It was demonstrated that both methods could successfully modify basalt fiber (BF) 3D woven fabrics. The 3D orthogonal woven composites (3DOWC) were produced with epoxy resin and 3D orthogonal woven fabrics as raw material by the VARTM molding process. The bending properties of the 3DOWC were tested and analyzed by experimental and finite element analysis methods. The results showed that the bending properties of the 3DOWC modified by KH570-MWCNTs and PDA were significantly improved, and the maximum bending loads were increased by 31.5% and 31.0%. The findings of the finite element simulation and the experiment results were in good agreement, and the simulation error value was 3.37%. The correctness of the finite element simulation results and the model’s validity further reveal the material’s damage situation and damage mechanism in the bending process.

## 1. Introduction

Three-dimensional textile composites have good delamination resistance, impact resistance, and dimensional stability [1], and are widely used in aerospace, wind turbine blades, marine industry, and automotive applications due to their advantages [2,3]. Composite materials are prepared by mixing two or more materials. At the critical position of reinforcement-matrix mixing, the interfacial properties of the composite are weak due to the poor combination of reinforcement and matrix. Therefore, high performance is given by changing the interfacial bonding ability of the composite materials [4].

With the advancement of science and the need for new high-performance materials at low cost, researchers are driven to modify current composite reinforcements or matrices. Multi-walled carbon nanotubes (MWCNTs) are potential candidates for interfacial modification of composites due to their excellent mechanical properties. Dopamine (DA), containing a large number of reactive functional groups, can adhere to any object surface after oxidative self-polymerization to produce polydopamine (PDA). Chen et al. [5] applied carboxylated carbon nanotubes to the basalt fiber (BF) surface to upgrade the mechanical properties of BF/vinyl ester resin (VE) composites. In addition, the mechanical properties of the modified composites, for instance, flexural rigidity, impact strength, and interlaminar fracture toughness, are improved. This indicated that carbon nanotubes’ high specific surface area provided an ideal interface for stress transferring. Lee et al. [6] explored the effect of carbon nanotube (CNT) modification on basalt/epoxy composites. Due to the nanotube incorporation made, the composite’s Young’s modulus and tensile strength were 60% and 34% higher than the unmodified composite’s, respectively, the thermal properties were also improved. This provided a basis for studying the influence of carbon nanotubes on the interfacial properties of the composites. Carbon nanotubes effectively improve the composites’ mechanical characteristics. However, CNTs have a robust and attractive force, which leads to mutual attraction resulting in the agglomeration and unwanted stress concentration. Therefore, dispersion becomes one of the most critical processes in using carbon nanotubes. Scholars have mainly used ultrasonic, stirred, mixed, and free arc dispersion [7,8,9,10] to disperse carbon nanotubes in polymers. However, researchers believe that functionalizing carbon nanotube surfaces can significantly enhance interfacial contact between carbon nanotubes, reinforcements, and the matrix [11,12]. Chen et al. [13] investigated the dispersion effect of four dispersants on carbon nanotubes and found that the surfactants formed bilayer adsorption tightly wrapped around the surface of MWCNTs under the impact of electrostatic gravitational force, hydrophobic force, and van der Waals force. Surfactants improved the dispersion of carbon nanotubes. In addition, this verified that the variation of functional groups on the surface of carbon nanotubes could effectively improve their dispersion performance. The results of this experiment provide the theoretical basis for this paper.

In addition, dopamine, a bio-based binder, exhibits exceptional benefits. Dopamine includes superior water solubility and excellent adherence to any substance through reactive groups (e.g., amino and catechol groups), mild reaction conditions for practical applications, low cost, and so on. The outstanding advantages make dopamine a reinforcing filler for interfacial reactions in composites. The use of polydopamine produced by the self-polymerization of dopamine improves the interaction between the reinforcement and the matrix, and it is a green and friendly modification method. Kim et al. [14] investigated the influence of polydopamine on the mechanical strength and stiffness of carbon and aramid composites. They found that surface treatment could improve the composite’s mechanical properties. Huan et al. [15] chemically modified the carbon fibers with polydopamine (PDA) to enhance the dispersibility and interfacial bonding strength with epoxy resin. The experimental results showed that adding polydopamine could effectively improve the mechanical properties of the composites, such as tensile strength, impact strength, and yield strength. Zhang et al. [16] investigated using polydopamine-modified glass fibers to prepare short glass fiber-reinforced polymer (GFRP) laminates. In addition, they found that polydopamine modification was a simple and effective method to significantly enhance the mechanical characteristics and the interfacial contact of GFRP laminates. Therefore, the modified composites with polydopamine are considered a relatively environmentally friendly and low-cost method.

The damage of fiber-reinforced composites usually consists of weak interfacial cracking between the fibers and the matrix. Then the interfacial cracking extends to matrix cracking and fiber pull-out. The surface of BF is relatively smooth and has weak interfacial bonding with the matrix material. BF composites are prone to fiber pull-out, fracture, and interfacial debonding when subjected to stress, and the mechanical properties are reduced. In order to enhance the mechanical properties of BF composites, the interfacial region of the composites must be modified first. Therefore, in this study, to improve the interfacial properties, surface modification with functionalized carboxylated carbon nanotubes (KH570-MWCNTs) and poly(dopamine) (PDA) was performed. FT-IR and SEM were used to characterize the modified fabrics. The unmodified, KH570-MWCNTs-modified, and PDA-modified 3D orthogonal woven fabrics with basalt filament yarns were used as the reinforcement, and bisphenol A epoxy resin was used as the matrix. The composites were prepared by VARTM. Experimental and finite element analysis methods were used to test and analyze the bending properties of the composites, and the load-displacement curves and failure mode were obtained and revealed the damage modes and mechanisms of the materials.

## 2. Materials and Methods

### 2.1. Materials and Equipment

Eight hundred tex basalt filament yarns (Zhejiang Shijin Co., Ltd., Dongyang, China); bisphenol A epoxy resin, curing agent, and promoters (Changzhou Lepang Composite Material Co., Ltd., Changzhou, China); multi-wall carbon nanotubes (Nanjing Xianfeng Nanotechnology Co., Ltd., Nanjing, China); 3-(isoprenyl oxygen) propyltrimethoxysilane KH570 (Wuhan KEMIK Bio-pharm Technology Co., Ltd., Wuhan, China); anhydrous ethanol (Guangzhou Kangben Biotechnology Co., Ltd., Gangzhou, China); ice acetic acid (Tianjin Guangfu Technology Development Co., Ltd., Tianjin, China); polydopamine (Wuhan KEMIK Bio-pharm Technology Co., Ltd., Wuhan, China); and hydrochloric acid (Tianjin Comio Chemical Reagent Co., Ltd., Tianjin, China) were used. A Universal Sample Making Machine (ZHY-W, Chengde Jiande Testing Instruments Co., Ltd., Chende, China); a Universal Material Testing Machine (TH-8102S, Cadiz Intelligent Measurement and Control Technology Co., Ltd., Suzhou, China); a Double Digital Display Magnetic Stirring (HJ-4B, Changzhou Jintan Jingdainstrument Manufacturing Co., Ltd., Changzhou, China); and an Ultrasonic Disperser (Scientz-2400F, Xinzhi Biotechnology Co., Ltd., Ningbo, China) were employed.

### 2.2. Sample Preparation

#### 2.2.1. Design and Weaving of 3D Orthogonal Woven Fabric with Basalt Filament Yarns

**Figure 1:**
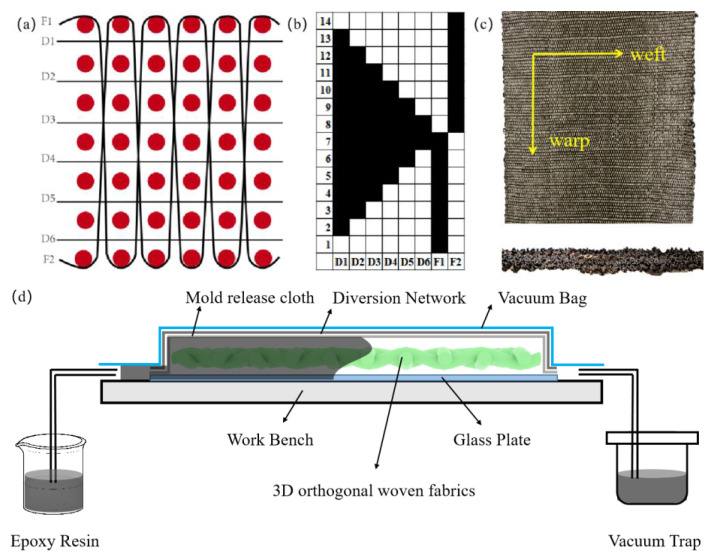
Structural pictures of the 3D orthogonal fabric: (**a**) warp cross-section diagram; (**b**) organizational chart; (**c**) 3D-orthogonal woven fabric; (**d**) VARTM.

Eight hundred tex basalt filament yarns were used as warp and weft yarn. After a good design, seven layers of 3D orthogonal woven fabric with basalt filament yarns were successfully woven on a loom. Figure 1 shows the structural pictures of the 3D-orthogonal woven fabric. Figure 1a is the warp cross-section diagram. In Figure 1a, the circles represent the weft yarns, the flexed lines represent the warp yarns, and the warp and weft yarns were well interwoven in the fabric. In Figure 1b, the organizational chart was drawn based on the warp cross-section diagram, with black representing the warp tissue points and white representing the weft tissue points. Figure 1c showed the completed 3D orthogonal woven fabric with basalt filament yarns. The weaving parameters are shown in Table 1.

#### 2.2.2. KH570-MWCNTs and PDA Modification

KH570-MWCNTs were used to prepare a carbon nanotube solution with distilled water and anhydrous ethanol as components (M distilled water + M anhydrous ethanol) × 1.05 = M (3D orthogonal woven fabric), where the volume ratio of anhydrous ethanol to distilled water was 4:1. A certain amount of anhydrous ethanol and distilled water were then measured, and the pH value of the anhydrous ethanol and distilled water solution was adjusted to 3.5–4.5 with ice acetic acid. the above KH570-MWCNTs were then weighed, and 0.1% of the weight of the basalt fiber 3D woven fabric was also weighed and added to the above solution. The KH570-MWCNTs solution was formed by stirring well with a glass rod and then ultrasonically dispersed for 1 h. The 3D orthogonal woven fabric was soaked in the KH570-MWCNTs solution for 24 h and dried at 120 °C to obtain the 3DOWC modified by KH570-MWCNTs.

Additionally, 10 mM/L Tris buffer with distilled water was prepared, and its pH was adjusted to 8.5 with hydrochloric acid, then 2 g of PDA was added to prepare the solution concentration to 2 g/L. Subsequently, the 3D orthogonal woven fabric was put into the PDA solution until wholly submerged, and the material was stirred in the PDA solution at room temperature using the rotor for 24 h. The 3D orthogonal woven fabric was then removed and dried at 80 °C for 12 h.

#### 2.2.3. Preparation of 3DOWC

The VARTM was used to prepare 3DOWC, as shown in Figure 1d. Two modified 3D orthogonal woven fabrics were used as reinforcement, and the bisphenol A epoxy resin mixture solution was added as matrix (bisphenol A epoxy resin with curing agent, V resin: V curing agent = 100:85). The VARTM was cured for 2 h at 90 °C, 1 h at 130 °C, and 4 h at 140 °C to obtain the modified 3DOWC with basalt filament yarns.

### 2.3. Characterization Methods and Bending Tests

To investigate the changes in the functional groups and surface morphology contained in the fabrics before and after modification, FT-IR and SEM were used to conduct microscopic tests on the unmodified KH570-MWCNTs and PDA-modified fabrics.

**Figure 2:**
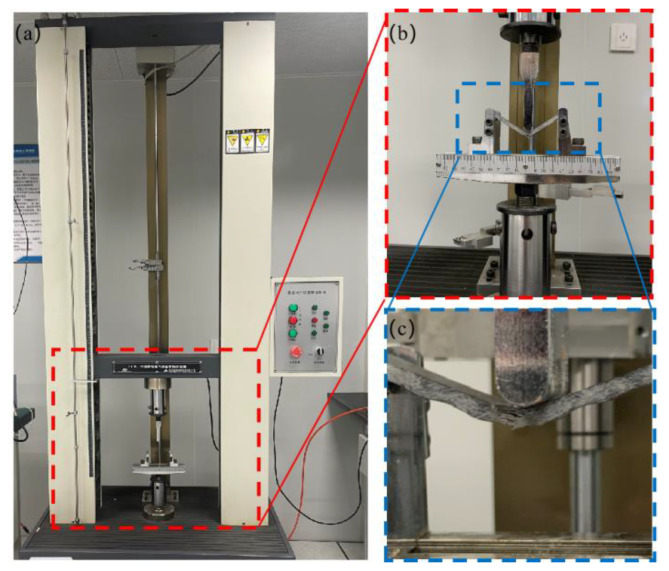
Test system: (**a**) testing equipment system; (**b**) bending test; (**c**) partial enlarged picture.

The warp and weft bending specimens of the unmodified and modified 3DOWC with basalt filament yarns were prepared for testing. As per the bending performance testing method for fiber-reinforced plastics outlined in GB/T 1449–2005, a three-point bending test was conducted on 100.00 mm × 15.00 mm × 4.30 mm specimens. The TH-8102S universal testing machine was utilized to conduct three-point bending experiments, and the test experiments were shown in Figure 2.

## 3. Results and Discussion

### 3.1. FT-IR Characterization

**Figure 3:**
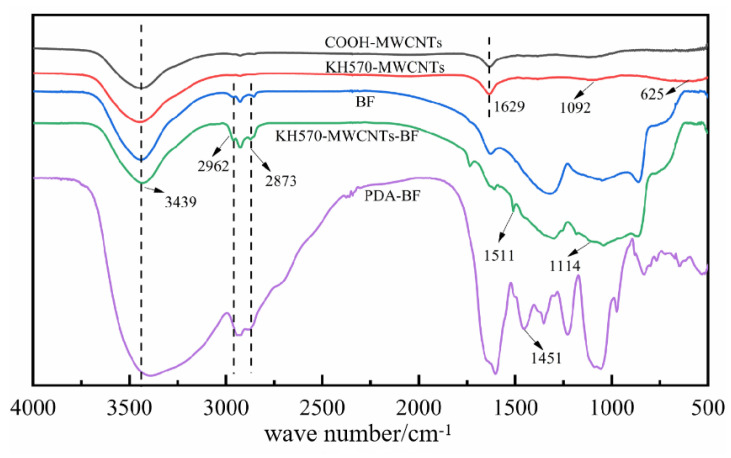
FT-IR of COOH-MWCNTs, KH570-MWCNTs, BF, KH570-MWCNTs-BF, PDA-BF.

FT-IR of COOH-MWCNTs, KH570-MWCNTs, BF, KH570-MWCNTs-BF, and PDA-BF was shown in Figure 3. From Figure 3, the FT-IR of COOH MWCNTs and KH570 MWCNTs, observations of stretching vibration peaks for -COOH and -OH at 3439 cm^−1^, and the vibration peak of C=C conjugated double bond at 1629 cm^−1^ were made [17], which indicated that there were substantial oxygenated functional groups including -OH, -COOH, and -COC in the carboxylated carbon nanotubes. After modification of coupling agent KH570, a telescopic vibration peak of Si-O-C [18] appeared at 1092 cm^−1^. Combined with the principle of COOH MWCNTs modified with KH570, it was found that the unmodified COOH-MWCNTs contained functional groups such as -COOH, -OH, and C-O-C. At the same time, the KH570 modification caused stretching vibrational peaks for Si-C and Si-O bonds and bending vibrational peaks for Si-O and C-O in the KH570-MWCNTs. The occurrence of these functional groups proved that KH570 successfully modified the COOH-MWCNTs.

According to the FT-IR of Figure 3, BF and KH 570-MWCNTs, the basalt fiber 3D fabric that underwent modification with KH570 MWCNTs exhibited stretching vibration peaks at 2962 cm^−1^ and 2873 cm^−1^ for the -CH_2_ of the coupling agent and C-H group of the alkyl groups. This was due to the modification of carbon nanotubes by the coupling agent KH570. The stretching vibration peaks were observed at 1114 cm^−1^ for Si-C and Si-O, along with the stretching vibration peak at 1511 cm^−1^ for the C=C bond. These stretching vibration peaks provide evidence of the successful grafting of CNT onto the surface of the basalt fibers. The stronger and broader absorption region at 864–1300 cm^−1^ was because more KH570 MWCNTs were on the surface of BF and combined with silicon hydroxyl groups on the surface of BF in the form of Si-O-Si bonds.

According to Figure 3, the FT-IR of BF and PDA-BF showed that the unmodified BF contained O-H and C-H stretching vibration peaks at 2962 cm^−1^ and 2873 cm^−1^. The characteristic peaks of the PDA-modified basalt fabric are significantly weaker here compared to the unmodified specimens [19]. This attenuating phenomenon proved the successful attachment of the PDA to the BF. Furthermore, many phenolic hydroxyl groups in the PDA attached to the surface of the BF formed a solid stretching vibration peak at 1451 cm^−1^ for the PDA-BF [20]. According to the principle of PDA-modified BF, it was found that phenolic hydroxyl groups and N-H bonds appeared on the surface of the modified BF, which proved that PDA had successfully modified the BF.

### 3.2. BF Surface Morphology

**Figure 4:**
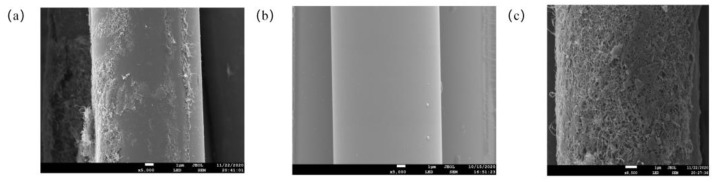
SEM of surface topography: (**a**) KH570-MWCNTs-BF; (**b**) BF; (**c**) PDA-BF.

The surface of the BF without modification was smooth except for a small amount of infiltrating agent [17], as shown in Figure 4a. In contrast, after KH570-MWCNTs modification, the surface of the BF became rough, as shown in Figure 4b. The PDA oxidation self-polymerization on the fabric surface produced a fold-like film tightly attached to the BF 3D woven fabric surface, thus increasing the BF surface roughness, as shown in Figure 4c.

### 3.3. Bending Property

#### 3.3.1. Load-Displacement Curve

**Figure 5:**
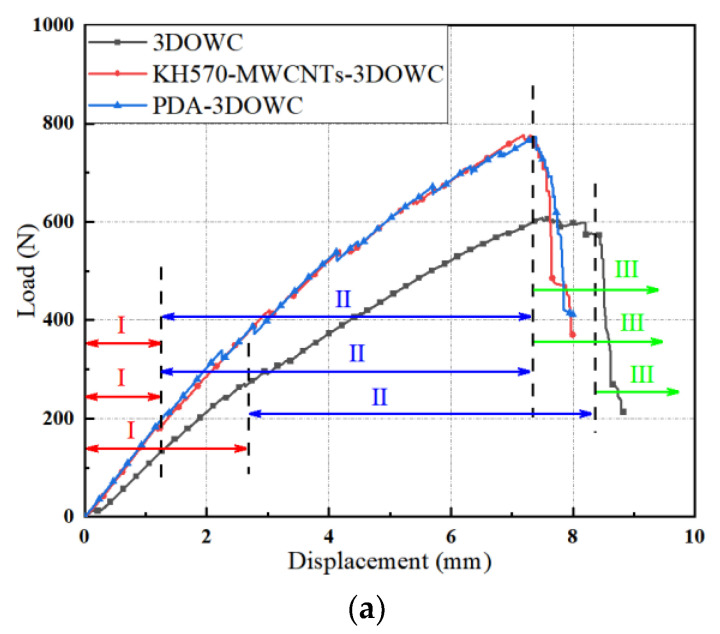

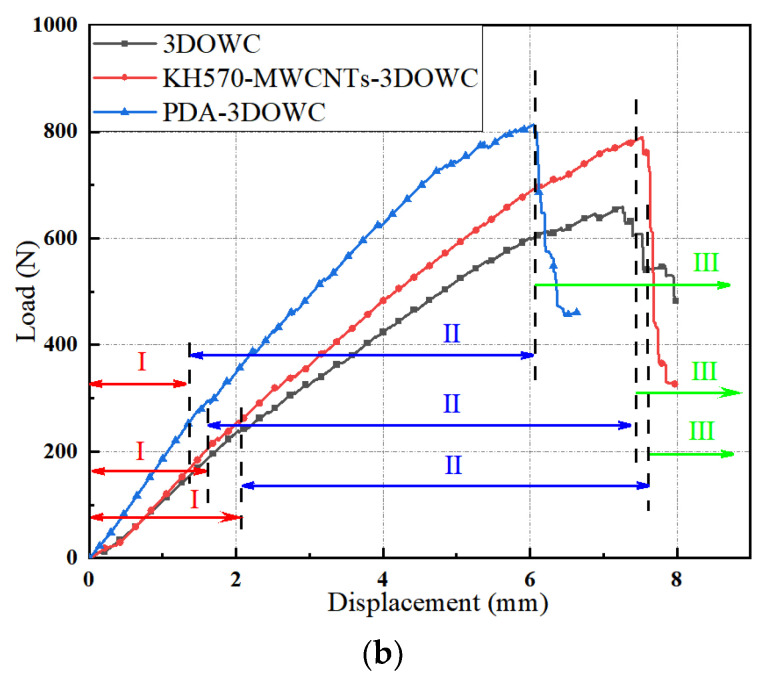
Bending load-displacement curves. (**a**) warp; (**b**) weft.

Figure 5a shows the warp bending load-displacement curves of 3DOWC, KH570-MWCNTs-3DOWC, and PDA-3DOWC. Figure 5b shows the weft bending load-displacement curves of 3DOWC, KH570-MWCNTs-3DOWC, and PDA-3DOWC.

Referring to Figure 5, the maximum bending load of 3DOWC modified by KH570-MWCNTs increased by 31.5% (warp) and 20.0% (weft) compared to the maximum bending load of unmodified 3DOWC. The maximum bending load of 3DOWC modified by PDA increased by 31.0% (warp) and 23.5% (weft). The specific tensile loads are shown in Table 2.

It was clear from Figure 5 that the load-displacement curves are divided essentially into three stages. As seen in Region I, with the continuous decline of the upper-pressure head, the composite material is subjected to a force on the whole. However, there are certain defects on the surface of the actual materials, so the test results were subjected to minor errors, and the load-displacement curves appear to rise approximately linearly. The reinforcement and the resin matrix were well combined, the composites did not show apparent damage because of the overall force, and the composites were in the elastic deformation stage. The second stage was Region II. As the load continued, the curves show a fluctuating rise. At this stage, the material’s surface exhibited a gradual emergence of micro-cracks, and the cracks were slowly transmitted. The gradual cracking of the matrix accompanied this, and the fibers were steadily pulled out until the maximum load was reached. The third stage was Region III, where the curves dropped rapidly, and the final brittle fracture of the material occurred.

#### 3.3.2. Bending Failure Mode

**Figure 6:**
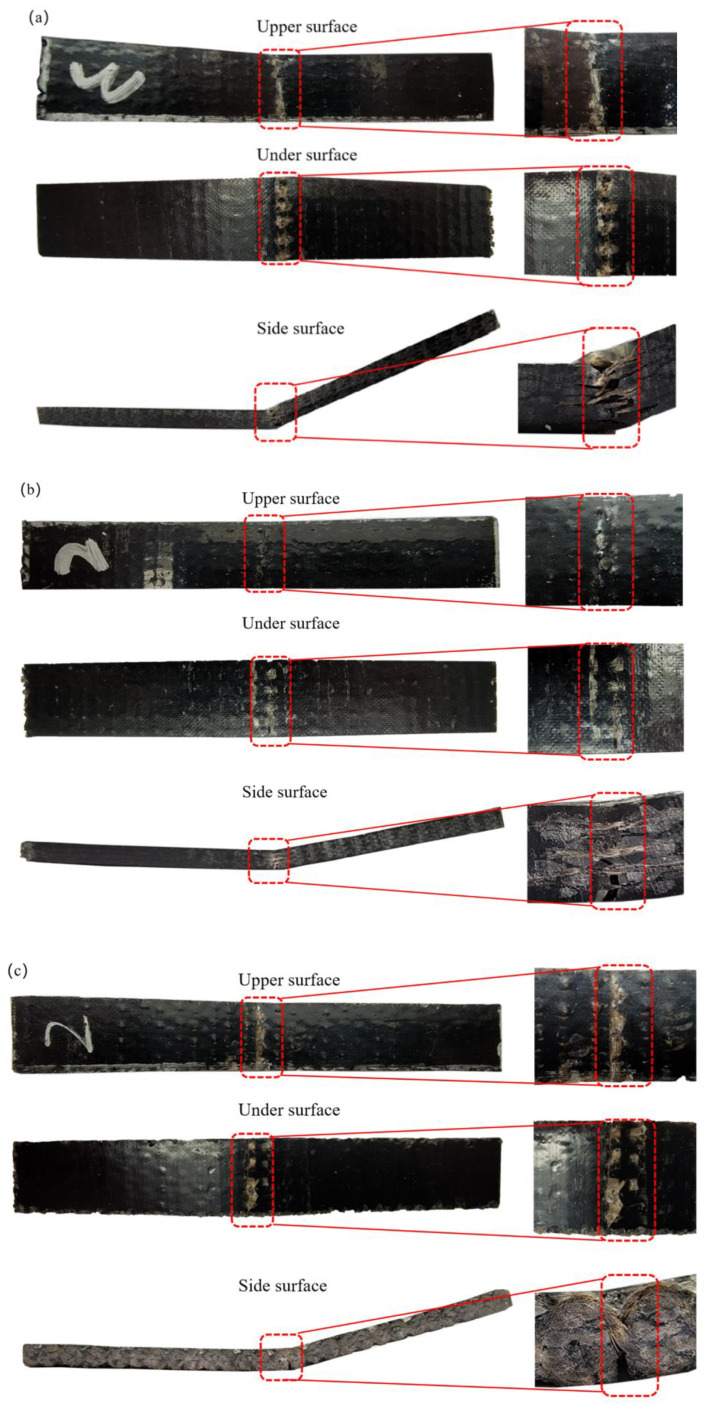
Bending failure mode: (**a**) 3DOWC; (**b**) KH570-MWCNTs-3DOWC; (**c**) PDA-3DOWC.

In order to further investigate the damage pattern and mechanism of 3DOWC, a Nikon Z6 II camera was used to analyze the damage morphology of materials, which is shown in Figure 6. Figure 6 is the bending failure modes of 3DOWC, KH570-MWCNTs-3DOWC, and PDA-3DOWC.

The final bending damage mode of the composite material is entirely yarn pull-out, fracture, yarn tensile damage, and debonding. MWCNTs can effectively promote load transfer and inhibit interface debonding, and rupture by acting as a “bridge” between BF and resin matrix. KH570-MWCNTs and PDA increase the surface roughness of basalt fibers and increase the interfacial friction between fibers and the resin matrix. BF were not easily pulled out when loaded, and the resin toughness was enhanced and not easy to crack. From Figure 6, it is found that load transfer was easy in unmodified composites due to the inadequate bonding at the interface between fibers and the matrix, the fiber bundle broke more seriously. The formation of stronger interfacial bonding between fibers and the matrix of KH570-MWCNTs-3DOWC and PDA-3DOWC inhibited the load transfer in the material. The matrix cracking at the fracture was weakened, and the fiber bundle fracture was improved. The interfacial bonding of the composites can be enhanced by KH570-MWCNTs and PDA. The modification of 3D orthogonal woven fabric with basalt filament yarns by KH570-MWCNTs and PDA was effective and successful.

### 3.4. Finite Element Analysis

KH570-MWCNTs-3DOWC were studied and modeled for finite element analysis.

#### 3.4.1. Material Properties

The different composition of each material leads to a significant difference in physical properties such as initial modulus, stiffness, and shear strength. Currently, basalt fiber generally includes 9 constant engineering parameters, mainly elastic and plastic. The resin matrix was only assigned Young’s modulus values. The parameters of yarn and resin were shown in Table 3.

#### 3.4.2. Mesh Model

**Figure 7:**
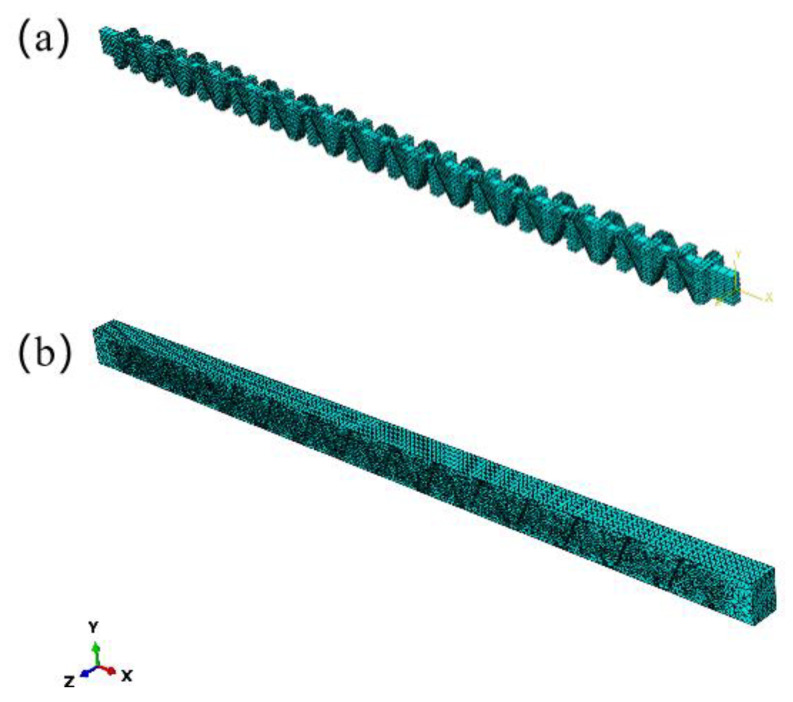
Mesh models: (**a**) yarn; (**b**) resin.

Mesh division is the most critical step of finite element model calculation, and a proper mesh can directly improve the accuracy and speed of finite element calculation. Therefore, KH570-MWCNTs-3DOWC was divided into several small cells. The stresses in each cell are calculated separately during subjection to bending load to obtain the overall stress-strain distribution of the composite. When meshing the model, the shape and number of mesh cells together determine the operation of the model calculation. A C3D8I hexahedral mesh was used to partition the yarn model with a regular shape, and a C3D4 tetrahedral mesh was used to partition the resin model with a complex cross-section shape. The mesh quantities of yarn and resin are shown in Table 4. The yarn model mesh partitioning diagram is shown in Figure 7a, and the resin model mesh partitioning diagram is shown in Figure 7b.

#### 3.4.3. Comparison of experimental and Finite Element Analysis Results

**Figure 8:**
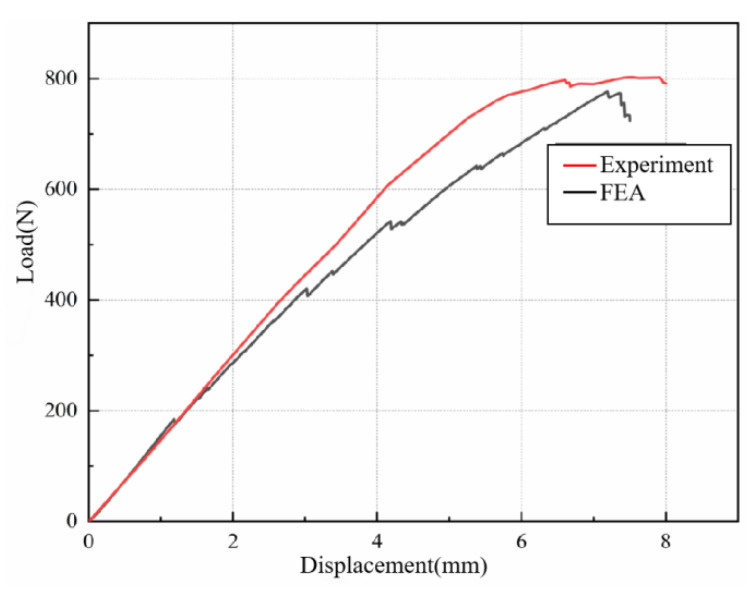
Load-displacement curves of KH570-MWCNTs-3DOWC of the experiment and FEA.

The load-displacement curves of KH570-MWCNTs-3DOWC of the experiment and FEA are shown in Figure 8.

The maximum bending load of KH570-MWCNTs-3DOWC measured during the experiment was 776.39 N, and the maximum bending load calculated through FEA was 802.53 N, with an error of 3.37%. The simulation outcomes showed a high level of consistency with the experimental results, affirming the accuracy of the finite element simulation and the soundness of the model. However, the finite element simulation made hypothetical conditions. The finite element simulation assumed that the various component materials in the composites were uniformly distributed, and the resin thoroughly soaked the yarn. The model and constraint conditions were approximately ideal. These perfect theoretical assumptions made the simulated curve smoother than the curve obtained from the experiment and made the simulation results slightly higher than the experimental results, with a certain degree of error between them.

#### 3.4.4. Analysis of Failure Mechanisms

**Figure 9:**
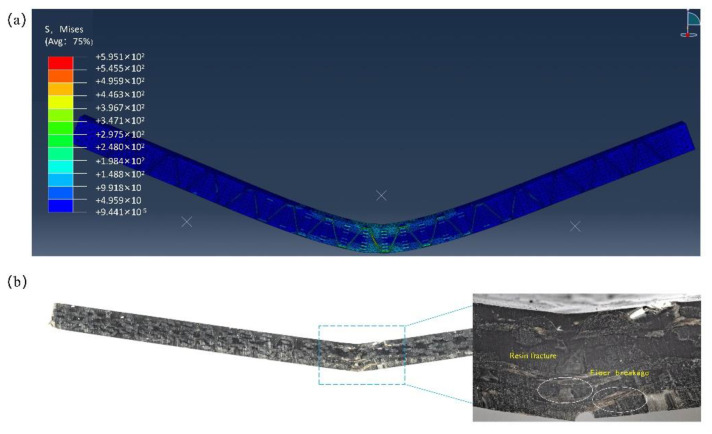
Final failure modes: (**a**) FEA; (**b**) experiment.

By observing the failure mode of the FEM model and the actual bending test sample under bending load, the overall failure of the composites, the damage behavior of the yarn under bending load, the degree and mode of resin damage, etc., were analyzed. The finite element simulation identified the ultimate failure mode of the material and experimental testing is shown in Figure 9.

**Figure 10:**
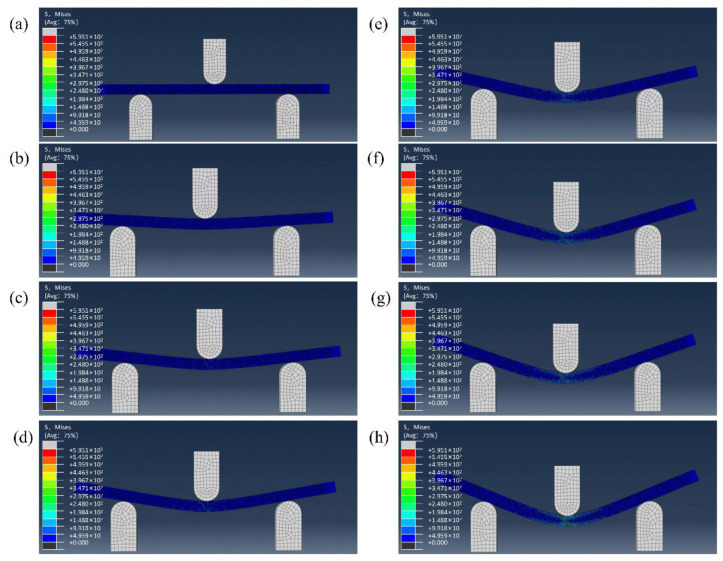
Stress cloud diagram: (**a**) 0; (**b**) 0.14; (**c**) 0.29; (**d**) 0.43; (**e**) 0.57; (**f**) 0.71; (**g**) 0.86; (**h**) 1.

**Figure 11:**
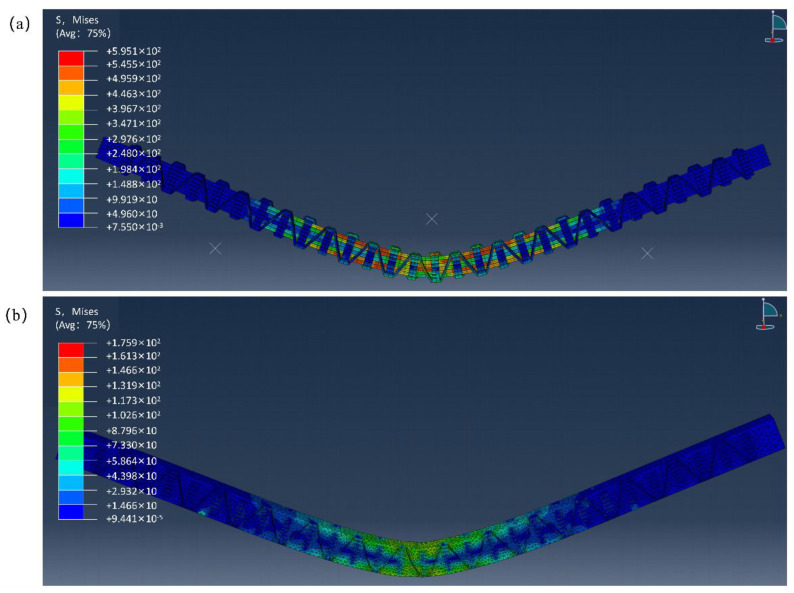
Failure diagrams: (**a**) yarn; (**b**) resin.

As seen in Figure 9, the experimental and simulated bending failure modes of KH570-MWCNTs-3DOWC were in good agreement. The material exhibited symmetrical bending deformation in the middle and protrudes outward. The upper pressing head caused stress concentration in the middle of the material, resulting in low-stress areas symmetrically distributed on both sides. The compression of the upper surface of the material caused it to be concave downward. In contrast, the under surface was subjected to the load’s transmission and the base’s support, causing fiber fracture and resin fragmentation. The entire material failure process could be obtained through finite element simulation. Figure 10 shows the stress cloud diagram of the composite material at the corresponding moments of 0, 0.14, 0.29, 0.43, 0.51, 0.71, 0.86, and 1 during cyclic loading. It was observed from Figure 10 that under bending load, the resin was deformation damaged, the fiber in contact with the upper pressing head was severely damaged, and the stress transmitted from the middle of the material to both ends. To further explore the stress-strain situation of the yarn and resin in the bending process, the yarn and resin models were separately observed, and their final failure diagrams were shown in Figure 11.

Figure 11a is the stress-strain distribution of the final failure state of the entire yarn model under Mise stress. It is seen from Figure 11 that the deformation area of the yarn model modified by KH570-MWCNTs was roughly consistent with the finite element simulation final failure diagram of the overall KH570-MWCNTs-3DOWC, and both showed a roughly symmetrical distribution to both sides from the upper clamping head. As the main load-bearing structure, the yarn bore more load than the resin, exhibiting more considerable bending stress. Consistent with the final failure diagram of the composite material in Figure 9a, local interfacial damage and shear fracture occurred in the yarn of the composite material. At the same time, significant local damage to the resin matrix also happened in the same position. Interface failure was caused by the accumulation of damage to the fibers and resin at the interface, which exacerbated the damage to the resin matrix in the middle of the material, as shown in Figure 11b.

From Figure 11b, it was seen that under bending load, the stress in the resin was mainly concentrated in the middle of the material, which was the reason for the yielding deformation of the material. As the resin bore a smaller load as a secondary load-bearing component, its relative modulus was lower than that of the yarn, so the resin was more prone to deformation under a bending load. Combined with Figure 11, it was found that the burden borne by the resin component was lower during the bending process. However, the elastic modulus of the yarn component was higher, so the load carried by the yarn was far higher than that of the resin.

Therefore, simulation results indicated that the yarn component played the central load-bearing role in the composite material. Its failure mode was consistent with the experimental results and mechanism. As the primary load-bearing component, the yarn only exhibited tension, deformation, and fracture. The yarn model and the material in the bending experiment did not show a layered phenomenon, indicating that the composite material has a solid anti-delamination ability and good integrity.

## 4. Conclusions

The main aim of the research was to enhance the mechanical properties of BF 3D woven composites by modifying BF 3D woven fabrics with KH570-MWCNTs and PDA. The research yielded the following conclusions:

FT-IR and SEM tests results showed that the surface roughness was increased after the change with KH570-MWCNTs and PDA. Substantial increase in surface roughness of BF were observed. Interfacial bonding was enhanced between reinforcement (BF 3D woven fabrics) and resin.

The bending properties of the 3DOWC, KH570-MWCNTs-3DOWC, and PDA-3DOWC were studied. The results showed that the maximum bending load of KH570-MWCNTs-3DOWC in the warp and weft directions was 776.39 N and 790.12 N, respectively, indicating an improvement of 31.5% and 20.0%. For PDA-3DOWC, the maximum bending load in the warp and weft directions was 773.26 N and 813.76 N, respectively, indicating an improvement of 31.0% and 23.5%.

The bending performance of the modified composite materials was improved. The primary failure mode during bending was the formation of cracks that extended downward due to bending compression in the resin and fiber on the upper surface and transverse cracks due to tensile stress on the lower surface that extended upward. The initial debonding crack propagated along the yarn path and transferred to the interface between the yarn and the matrix. Finally, the warp and weft yarns at the bottom were completely pulled out from the matrix, resulting in the bending failure of the sample.

Finally, a method combining micro finite element simulation with experimental results was used to compare and analyze the bending failure modes and mechanism. The simulation results showed good consistency with the experimental results. The simulation error value was 3.37%, proving the correctness and validity of the finite element simulation results and further revealing the material’s damage situation and mechanism during bending.

## Figures and Tables

**Table 1:** Weaving parameters.

	Linear Density/Tex	Thickness/ mm	Weaving Density (Yarn/10 cm)		Total Number of Warp Roots/(Roots)
	Warp/ Weft Yarns		Weft Density	Warp Density	
3D orthogonal woven fabrics	800/800	4.31	238	240	720

**Table 2:** Bending loads of different modified 3DOWC.

	Warp	Weft
3DOWC	590.85 N	658.52 N
KH570-MWCNTs-3DOWC	776.39 N	790.12 N
Increase rate	31.5%	20.0%
PDA-3DOWC	773.26 N	813.76 N
Increase rate	31.0%	23.5%

**Table 3:** Parameters of yarn and resin.

	E_11_/ GPa	E_22_/ GPa	E_33_/ GPa	Em/ GPa	V_12_	V_13_	V_23_	G_12_/ GPa	G_13_/ GPa	G_23_/ GPa
Yarn	60	12	12	-	0.28	0.28	0.25	23	23	4.0
Resin	-	-	-	2.6	-	-	-	-	-	-

Note: E11: The longitudinal elastic modulus of the composite material. E22 and E33: The transverse elastic modulus of the material. V12 and V13: Longitudinal Poisson’s ratio of composite materials. V23: The transverse Poisson’s ratio of the material. G12 and G13: Longitudinal shear modulus of composites. G23: The transverse shear modulus of the material.

**Table 4:** Number of units in FEA.

Projects	Mesh Quantities
Yarn	37,797
Resin	88,805

## Data Availability

The raw/processed data required to reproduce these findings cannot be shared at this time as the data also form part of an ongoing study.
